# Physical Treatments and Therapies for Androgenetic Alopecia

**DOI:** 10.3390/jcm13154534

**Published:** 2024-08-02

**Authors:** Siddhi Bianca Camila Lama, Luis Alfonso Pérez-González, Mehmet A. Kosoglu, Robert Dennis, Daniel Ortega-Quijano

**Affiliations:** 1 StimuSIL, Inc., Wilmington, DE 19802, USA; mehmet@stimusil.com; 2 Dermatology Department, University Hospital Ramón y Cajal, 28034 Madrid, Spain; pg.l.alfonso@gmail.com (L.A.P.-G.); danielortegaquijano@gmail.com (D.O.-Q.); 3 Biomedical Engineering Departments, UNC Chapel Hill and NC State University, Raleigh, NC 27695, USA; bob@bobslab.com; 4 Hair Disorders Unit, Grupo Pedro Jaén, 28006 Madrid, Spain

**Keywords:** androgenetic alopecia, hair loss, microneedling, laser hair therapy, PEMF, fractional lasers, nonablative lasers, LLLT, photobiomodulation, multimodal treatments

## Abstract

Androgenetic alopecia, the most common cause of hair loss affecting both men and women, is typically treated using pharmaceutical options, such as minoxidil and finasteride. While these medications work for many individuals, they are not suitable options for all. To date, the only non-pharmaceutical option that the United States Food and Drug Administration has cleared as a treatment for androgenetic alopecia is low-level laser therapy (LLLT). Numerous clinical trials utilizing LLLT devices of various types are available. However, a myriad of other physical treatments for this form of hair loss have been reported in the literature. This review evaluated the effectiveness of microneedling, pulsed electromagnetic field (PEMF) therapy, low-level laser therapy (LLLT), fractional laser therapy, and nonablative laser therapy for the treatment of androgenetic alopecia (AGA). It also explores the potential of multimodal treatments combining these physical therapies. The majority of evidence in the literature supports LLLT as a physical therapy for androgenetic alopecia. However, other physical treatments, such as nonablative laser treatments, and multimodal approaches, such as PEMF-LLLT, seem to have the potential to be equally or more promising and merit further exploration.

## 1. Introduction

Androgenetic alopecia (AGA) is the most common cause of hair loss, potentially affecting over 50% men and women throughout their lives. It is a non-scarring alopecia in which the progressive miniaturization of hair follicles leads to a loss of hair density. Without treatment, AGA may result in extensive areas of complete baldness, resulting in significant emotional repercussions for the patients and impacting their quality of life [1,2].

Currently, the treatment of AGA relies on the use of two fundamental drugs: minoxidil and 5-alpha reductase inhibitors (5ARIs). Minoxidil has been approved for this indication in topical form (2% or 5% concentrations), or is orally administered off-label (from 0.5 to 10 mg per day). 5ARIs include medications such as 1 mg finasteride, which is formally approved for this indication, and dutasteride, which is more effective than finasteride, but, to date, lacks formal approval in the vast majority of countries. These drugs have demonstrated satisfactory results in a significant percentage of patients with AGA [3,4,5,6,7].

However, certain limitations, such as the inability to use 5ARIs for patients with a history of hormone-dependent neoplasms or those desiring pregnancy, as well as the desire to improve therapeutic outcomes without continuously using pharmacological treatments, have led to the exploration of other treatment approaches, especially those based on physical therapies. With increasing evidence of their effectiveness and safety, these therapeutics represent a valid alternative and are positioning themselves as complementary approaches to improve the results achieved with conventional treatments in a significant number of patients [8,9].

## 2. Methods

This is a narrative review. Clinical pictures of male and female patients with androgenetic alopecia are presented, reporting the course of the disease, differential diagnoses, and common treatment options. This narrative presentation focuses on physical therapeutic options for the treatment of androgenetic alopecia, which can be divided into three categories: microneedling, laser hair therapy, and pulsed electromagnetic field therapy.

The available literature on androgenetic alopecia was analyzed, seeking non-pharmacological, nutraceutical, or regenerative medicine approaches to the treatment of this form of hair loss. A literature search was conducted using MEDLINE via PubMed and Google Scholar databases, covering the period from 1 January 1980 to 1 May 2024. The following keywords were used: androgenetic alopecia, pattern hair loss, microneedling, laser, LLLT, pulsed electromagnetic, pulsed electrostatic, pulsed electrical, and PEMF. Studies on other forms of hair loss were excluded unless they made specific mention of androgenetic alopecia as well. The exclusion criteria were conference abstracts and articles in languages other than English, Spanish, or Portuguese.

## 3. Results

### 3.1. Low-Level Laser Therapy (LLLT)

LLLT for AGA is based on the principle of photobiomodulation, a branch of photobiology that studies the effect of low-energy light on dysregulated biological processes. One of the primary underlying principles of photobiomodulation is hormesis, the use of low-level energy to stimulate a specific biological process, whereas the use of the same type of energy at higher doses could have a completely opposite, suppressive effect [10,11,12,13].

**Figure 1:**
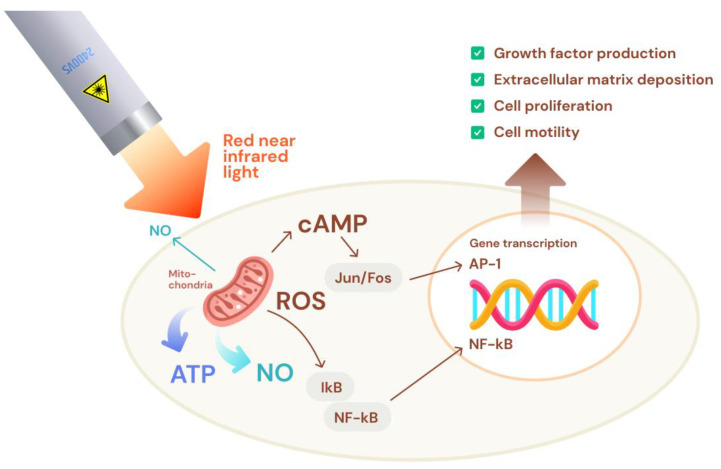
The effect of photobiomodulation on mitochondria and gene expression.

**Figure 2:**
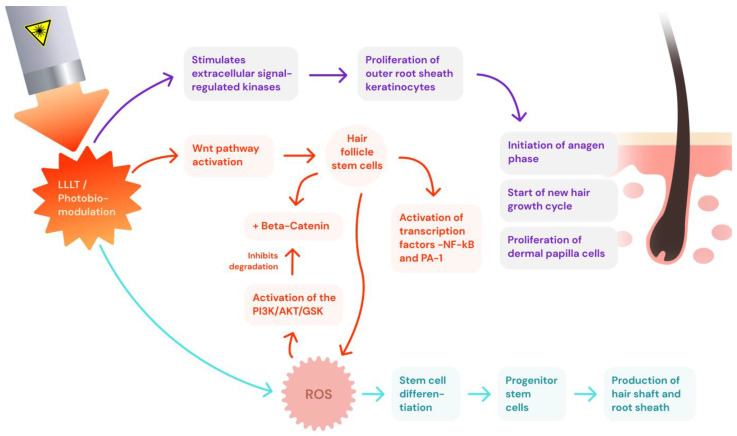
The pathways photobiomodulation use to influence hair growth.

The main mechanisms of action attributed to photobiomodulation involve the mobilization of molecules, such as reactive oxygen species (ROS), nitric oxide (NO), and adenosine triphosphate (ATP), as shown in Figure 1. This occurs through interaction with mitochondrial chromophores like cytochrome C oxidase (CCO), leading to mitochondrial respiration and ATP production. These molecules, including ROS, ATP, and NO, activate the redox-related signaling pathways, positively impacting hair growth by stimulating cellular proliferation, migration, and the differentiation of hair bulge stem cells, as shown in Figure 2. Additionally, ATP prompts increased hair growth through the transition from the telogen to anagen phases. Photobiomodulation also mediates skin benefits through pathways, such as VEGF and Wnt/β-catenin signaling, further enhancing its therapeutic effects [14,15,16]. In 2007, the United States Food and Drug Administration (FDA) cleared the first LLLT device, the Hairmax Laser Comb^®^, for male AGA. The device was cleared for the treatment of female pattern hair loss shortly after in 2011 [17,18,19]. There are, to date, over 80 LLLT devices that have been granted 510(k) clearance by the US FDA. These devices, which are generally designed as comb-type or helmet-type tools, typically have red laser diodes or red light-emitting diodes with wavelengths between 630 and 660 nm. Despite the difference in the number of diodes and the variations in wavelengths, power, and treatment times, these devices are considered to be nearly equivalent in effectiveness, with laser helmets showing slightly more effectiveness than laser combs or brushes [18,20,21].

Since then, several clinical trials have been conducted to demonstrate the efficacy of LLLT in the treatment of AGA, as shown in Table 1. These studies typically utilize short treatment times from around 8 to 30 min in duration, with treatments applied as infrequently as twice a week or as frequently as every day. Interestingly, the shortest treatment time of 8 min a day, three times per week over 26 weeks reported the largest increase: 25.7 hairs per cm^2^. Conversely, a 24 week treatment applying 30-min-a-day treatments produced an increase of just 6 hairs per cm^2^ [22,23,24,25].

In most LLLT studies, mild-to-moderate efficacy has been demonstrated showing improved hair density and tensile strength and reduced hair loss. While some studies did not report a clear overall improvement, more than 70% of patients expressed satisfaction with the results obtained, highlighting the absence of serious adverse effects and excellent treatment tolerance [22,23,24,26,27,28,29,30].

**Table 1:** Reported improvements in hair growth at 12–36 weeks following the administration of physical therapy.

Study	AGA Diagnosis	Treatment Regimen	Treatment	Subject Demographics	Duration (Weeks)	Improvement in Hair Density (Total Hairs/cm^2^)
Kumar et al., 2018 [31]		2× daily treatment	Minoxidil 5%	34 M 27.53 Y (avg.)	12	1.89 ± 8.94
	NH III-IV	2× daily treatment, as well as 8 sessions: four 1× per week, then four fortnightly sessions	Minoxidil 5% Microneedling	34 M 24.56 Y (avg.)	12	12.82 ± 6.82
Dhurat et al., 2013 [32]		2× daily treatment	Minoxidil 5%	44 M 28.6 Y (avg.)	12	22
	NH III-IV	2× daily treatment, as well as 1× weekly microneedling (with no minoxidil on day of microneedling)	Minoxidil 5% Microneedling	50 M 28.6 Y (avg.)	12	91.4
Choi and Park 2022 [33]	NH II+ L I+	15 min LLLT and 10 min PEMF 17 treatments: 1× per week for 12 weeks, then 1× every 2 weeks for 8 weeks, then 1× in 4 weeks	PEMF and LLLT	31 M, 9 F 40.18 ± 6.77 Y	23	24.8 ± 2.65
			Sham	28 M, 12 F 39.55 ± 7.75 Y	23	6.5 ± 1.85
Bureau et al., 2003 [34]	NH I-VII L I-II	30 min 3× per week	PEMF and Nutraceutical	31 M, 9 F 38.6 ± 8.14 Y	26	34
			Sham and placebo nutraceutical	21 M, 8 F 40.6 ± 9.32 Y	26	9
Maddin et al., 1990 [35]		12 min 20 1× per week 4 2× per week	PEMF using Electrotricho-genesis	30 M 37.23 ± 5.24 Y	12	3.67 ± 6.72 *
			PEMF using Electrotricho-genesis	30 M 37.23 ± 5.24 Y	24	7.08 ± 8.59 *
	NH III-IV		PEMF using Electrotricho-genesis	30 M 37.23 ± 5.24 Y	36	11.87 ± 10.61 *
			Sham	26 M 37.96 ± 6.76 Y	12	−1.97 ± 8.79 *
			Sham	26 M 37.96 ± 6.76 Y	24	1.93 ± 8.3 *
			Sham	26 M 37.96 ± 6.76 Y	36	5.61 ± 10.25 *
Maddin et al., 1992 [36]	NH III-IV	12 min 20 1× per week 4 2× per week	PEMF using Electrotricho-genesis	14 M 37 ± 2.1 Y	24	11.66 ± 1.38 *
		12 min 26 1× per week 4 2× per week	PEMF using Electrotricho-genesis	14 M 37 ± 2.1 Y	30	17.59 ± 1.98 *
Leavitt et al., 2009 [22]	NH IIa-V FS I-IV	15 min 3× per week	LLLT using the HairMax LaserComb	71 M 47.9 ± 8.7 Y	26	17.3 ± 11.9 *
			Sham	39 M 47.9 ± 8.7 Y	26	−8.9 ± 11.7 *
Kim et al., 2013 [23]	NH III-VII L I-III	18 min daily	LLLT using the Oaze 3R Helmet	15 M and F 43.9 ± 12.2 Y	24	17.2 ± 12.1
			Sham	14 M and F 44.5 ± 11.4 Y	24	−2.1 ± 18.3
Mai-Yi Fan et al., 2018 [25]	NH IIa-V LI-4, II-1, II-2 FS I-IV	30 min 3× per week	LLLT using the Restore ID-520	61 M 37.2 ± 8.3 Y 13 F 37.2 ± 8.3 Y 61 M 37.1 ± 8.1 Y	24 24	6 ± 12.5
			Sham	13 F 37.1 ± 8.1 Y		−2 ± 12.6
		11 min 3× per week	LLLT using 9-beam HairMax LaserComb	42 F 49.3 ± 9.1 Y	26	20.2 ± 11.2 *
			Sham	21 F 49.8 ± 7.3 Y	26	2.8 ± 16.5 *
		8 min 3× per week	LLLT using 12-beam HairMax LaserComb	39 F 48.7 ± 10.2 Y	26	20.6 ± 11.6 *
Jimenez et al., 2014 [24]	NH IIa-V		Sham	18 F 49.1 ± 8.3 Y	26	3.0 ± 9.3 *
		15 min 3× per week	LLLT using 7-beam HairMax LaserComb	24 M 47.8 ± 9.0 Y	26	18.4 ± 13.7 *
			Sham	14 M 40.9 ± 9.5 Y	26	3.0 ± 9.3 *
		11 min, 3× per week 8 min, 3× per week	LLLT using 9-beam or HairMax Laser Comb or 12-beam HairMax Laser Comb	21 M 45.6 ± 9.3 Y 19 M 47.9 ± 9.6 Y	26	20.9 ± 13.5 * 25.7 ± 17.1 *
			Sham	21 M 45.9 ± 10.4 Y	26	9.4 ± 12.9 *
Lodi et al., 2021 [37]	NH III-VI	24 min 2× per week	Blue LLLT	20 M 38.55 ±11.68 Y	10	11 ± 3
Lee et al., 2011 [38]	L I-II	10 treatments (every fortnight)	1550 fractional Er/Glass Laser	27 F 41.8 ± 1.96 Y	20	57 ± 14

Abbreviations: NH = Norwood–Hamilton Scale; FS = Fitzpatrick Scale; L = Ludwig Scale, M = Male; F = Female; Y = Years of age. *: Only ‘terminal hairs per cm/2′ were reported for this study.

Adverse effects from LLLT used as an AGA treatment are usually very limited, including mild adverse events, such as scalp tenderness and itchiness. The side effects are generally self-resolvable within a 2 week period, and no serious side effects requiring the discontinuation of treatment have been reported [20]. However, as an increasing number of copycat devices entered the market, manufacturers attempted to increase the effectiveness of their LLLT devices by changing the number of diodes, the power emitted, and other variables. Manufacturers like LaserCap noted that increasing power correlates with increased effectiveness, but it can also result in increased adverse events [39].

FDA-cleared LLLT devices usually deliver less than 1 J/cm^2^ of fluence (light energy per area at the surface) at the skin’s surface [39]. But due to losses in the epidermis, around 95% of the incident irradiance does not reach 2 mm depth [40]. When some laser cap manufacturers tried to increase light power, and thus fluence, in order to increase effectiveness, it led to adverse events, such as burns, itchiness, and hair shedding, which resulted in warning letters from the US FDA [39].

As the effects of photobiomodulation are independent of temperature increase, it is neither necessary nor desirable to use excessively high temperatures that could cause epidermal damage. Moreover, the use of excessive energies could result in a suppressive effect due to the hormetic behavior of photobiomodulation. Safely increasing the effectiveness of photobiomodulation treatments is essential in order to improve hair regrowth results.

### 3.2. Alternative Forms of Laser Hair Therapy

Although LLLT remains the only type of light and laser therapy that has been approved as a treatment for AGA, other light and laser therapies are being assessed in clinical trials and used privately in dermatological clinics. The clinical studies have reported a number of positive improvements on skin and hair, with a handful of case studies reporting unique secondary effects, such as melanogenesis.

#### 3.2.1. Fractional Lasers

Multiple fractional lasers have been used to treat AGA, including 1550 nm fractional erbium–glass lasers, fractional CO_2_ lasers, Er/YAG fractional lasers, and fractional thulium lasers [41]. When used properly, fractional lasers are ablative, but cause minimal damage. They strike a happy medium between nonablative and ablative lasers, acting only on the stratum corneum, the outermost segment of the skin’s surface, and causing microtrauma. However, fractional lasers can cause thermal damage zones within skin that can kill some hair follicles and damage some of the surrounding tissue [42]. The microscopic injuries induced by fractional lasers can trigger a wound healing environment that stimulates a regenerative wound healing process, activates hair follicle cells, and results in hair growth. Simultaneously, the microtrauma specific to the stratum corneum allows for the enhanced transdermal delivery of drugs and topical nutraceuticals to the scalp’s hair follicles [43].

Fractional lasers are currently used for a number of dermatological purposes, including the treatment of pigmentation-related issues, scars, and wrinkles [44,45,46]. They are also being explored as solutions for the treatment of other types of hair loss, including scarring alopecia [47]. More recently, there has been increased interest in the use of fractional ablative lasers to facilitate the delivery of topical treatments (i.e., laser-assisted drug delivery), such as minoxidil, platelet-rich plasma, and various growth factors, thereby enhancing their penetration and efficacy [43,48,49]. However, fractional lasers have not been yet approved as treatments for any type of alopecia, and it is likely that their role in the treatment of alopecia will evolve as a complement to enhance the administration and efficacy of the standard treatments [42].

#### 3.2.2. Nonablative Lasers

Unlike fractional lasers and ablative lasers, nonablative lasers do not intentionally damage any component of the skin. However, nonablative lasers still deliver heat, unlike FDA-cleared LLLT for the treatment of AGA (that is sometimes referred to as a “cold laser”). A variety of nonablative lasers are used in dermatology, but erbium–glass (Er/G) is the primary nonablative option used in the treatment of AGA. Er/YAG lasers have also shown promise in the treatment of this form of hair loss [41,50].

The nonablative laser uses light that is absorbed in the most superficial layer of the skin, while releasing heat that diffuses into the skin’s deeper layers. The heat penetrates up to 0.5 mm through the skin, triggering paracrine signaling, activating fibroblasts, and prompting a regenerative response. This treatment can be used as an AGA monotherapy for men and women. Recently, ten Er/G treatments on a small group of women with AGA showed an improvement of 57 total hairs/cm^2^ after 5 months [38]. Nonablative Er/G and Er/YAG treatments have also been used in conjunction with platelet-rich plasma therapy, minoxidil, and oral supplements [41,50].

#### 3.2.3. Blue and Yellow LLLT

Photobiomodulation using blue light and yellow light have also been used in the treatment of AGA. Blue light, which is typically administered at wavelengths between 380 nm and 460 nm, is typically used for the management of cutaneous diseases and wound healing. It has also been reported to have anti-inflammatory and antimicrobial effects when applied to skin [51]. At 453 nm, blue light has been shown to stimulate the cryptochrome (CRY)-1 protein in keratinocyte cells, which helps regulate hair follicle cell proliferation and the maintenance of the anagen phase of the hair growth cycle [52].

Recently, a 10-week study administering blue LED light (at 417 ± 10 nm with a fluence of 120 J/cm^2^ and power intensity of 60 mW/cm^2^ ± 20%) reported an increase in 90% of their patients’ hair density and hair shaft diameters. The treatments in this study were performed twice a week, as shown in Table 1. Notably, the study also reported the darkening of 30% of the participants’ hair [37].

Blue light has also been used in combination with red light as part of a treatment regimen for skin issues like acne and to restore hair growth. When treating acne, the combination of blue and red light has been shown to reduce inflammatory and non-inflammatory acne, decrease the size of the sebaceous gland, and consequently reducing sebum output [53]. When used for hair loss, blue light has also been used in combination with red light, along with microneedling and growth, in non-responders to finasteride. In this study, blue light (423 nm) was specifically thought to reduce scalp sebaceous glands and scalp fat and activate keratin in hair shafts. While this study was successful in improving hair regrowth, it combined too many variables to be able to attribute any specific improvements to the inclusion of blue photobiomodulation [54].

Compared to other colors of light used in photobiomodulation, yellow light is used sparingly. However, one recent study utilized a combination of dissolvable microneedle patches containing exosomes and yellow light irradiation (intended to reduce inflammation) to improve hair regrowth. Although this is the first study to report the use of yellow light photobiomodulation for this purpose, it may indicate that there is the potential for this form of light therapy to be useful in the treatment of alopecia [55].

### 3.3. Pulsed Electromagnetic Field Therapy (PEMF)

Pulsed electromagnetic field therapy (PEMF) devices are well-established tools for bone healing, wound healing, and pain relief, with the earliest devices dating back to the 1950s. PEMF is also known to have a positive effect on skin and hair cells and has been shown to promote hair regrowth. Electromagnetic fields are known to open calcium channels and facilitate the entrance of calcium ions. This mechanism of action is thought to be the fundamental principle behind all the PEMF devices used in regenerative medicine. The modulation of ion channels in this manner prompts cell differentiation and communication, as has been shown on dermal papilla mesenchymal cells in vitro due to increased MAP2 or Nestin gene expression [56]. PEMF also directly stimulates the drivers of hair restoration, promoting hair shaft growth and increasing hair length. It works by upregulating enzymes and increasing expression of cytokines and proteins, particularly those involved in the Wnt/β-catenin signaling pathway [57].

In vivo, PEMF encourages hair follicle regrowth, accelerates the hair growth rate, and can even promote hair follicle regeneration. Epidermal stem cells and dermal papilla cells were injected into the epidermis of mice, and their skin was then exposed to electromagnetic fields, facilitating the induction of hair follicle regeneration. The combined treatment specifically resulted in a higher density of hair follicles, implying that PEMF would complement hair transplants and other regenerative biological therapeutics for hair loss, such as exosomes, growth factors, stem cell, and platelet-rich plasma treatments [58,59].

However, the mechanism of action of PEMF in relation to AGA is likely quite complex, as it is also known to reduce the stressors, modulate inflammation, and indirectly balance hormone production and the melatonin levels in the neuroendocrine system. All four of these major factors have been implicated in the progression and exacerbation of AGA [31,60,61,62,63,64]. The observed effects of PEMF on such a wide range of body systems is presumably due to the fact that the cell membrane is the main target for PEMF signals. However, even the well-established anti-inflammatory effect of PEMF arises from a mechanism of action that has yet to be fully elucidated, and is currently believed to be partially due to PEMF’s ability to stabilize membranes and restore faulty Ca2+ ATPase and intracellular Ca2+ levels, inhibiting prostaglandin E2 biosynthesis [60].

#### 3.3.1. PEMF Treatments for AGA

The pioneering work that led to the use of electrical fields to treat AGA was inspired by an anecdotal report of hair regrowth following transcutaneous electrical neural stimulation (TENS). The TENS device reduced hair shedding and improved the hair texture and hair growth rate in four men. In the 1980s, another two studies showed that PEMF could also counteract hair loss, with 84% of a group of 25 subjects and 70% of a group of 40 subjects showing improvements in hair regrowth after using an early version of a PEMF device [35].

Maddin et al. pioneered the first two formal studies of PEMF for hair loss in the 1990s. Their success set the stage for the use of PEMF as a treatment for AGA. In his first study, Maddin reported an increase of 7 terminal hairs/cm^2^ vs. the control group increase of 2 hairs/cm^2^ after 24 weeks, and an increase of 12 terminal hairs/cm^2^ after 36 weeks vs. the control group increase of 6 terminal hairs/cm^2^, as shown in Table 1. This was equivalent to an increase in the terminal hair count of over 66% after 36 weeks of treatment compared to the control group’s increase of 26%. Notably, 83% of the subjects saw an increase in hair growth, while 97% exhibited regrowth or no further hair loss [35].

The longitudinal data for a part of the same study group were reported in a second extension study, which found that terminal hair counts continued to increase to 19 terminal hairs/cm^2^ at 52 weeks and 38 terminal hairs/cm^2^ at 70 weeks. This showed that PEMF was able to produce continuous improvement in hair regrowth, even after more than a year of treatment [35,36].

#### 3.3.2. Multimodal PEMF Treatments for AGA

Since the 1990s, AGA in males and females has been treated using PEMF, but only in combination with another treatment. Bureau et al. used PEMF in combination with essential plant oils administered over 26 weeks. A total of 83% of the treatment group exhibited a decrease in hair loss, while 53% of patients had an increase in hair count of 20% or more. The combination of their topical solution with PEMF resulted in an increase of 34 total hairs/cm^2^ compared to 9 hairs/cm^2^ in the control group, which used a placebo nutraceutical with anti-inflammatory properties and a sham PEMF device [34].

More recently, Choi and Park combined LLLT and PEMF over a 24-week period to treat androgenetic alopecia in men and women. The use of PEMF with LLLT resulted in an increase of 25 total hairs/cm^2^ vs. 6.5 total hairs/cm^2^ in the sham device control. The side effects were limited to minor issues, such as itching, burning, redness, and irritation [33].

Despite overwhelmingly positive results, the data on the use of PEMF as a treatment for AGA are still limited. To date, less than half a dozen papers have been published studying PEMF as a hair loss treatment. An established mechanism of action, defined parameters, recommended treatment regimens, and longitudinal data are lacking.

### 3.4. Microneedling

Microneedling is a minimally invasive procedure that is often used for cosmetic and aesthetic dermatological indications, such as the removal or minimization of acne scars, wrinkles around the eyes and mouth, skin laxity, scars from burns or trauma, and stretch marks. More recently, it has also been used as both a monotherapy and adjunct hair loss treatment as it can contribute to the induction of new hair growth cycles and the production of new hair strands. The wound healing cascade that it triggers increases blood flow and releases growth factors that help create a conducive environment for hair regeneration. Microneedling for hair loss involves the use of multiple fine needles, usually in the form of a dermaroller or dermapen-type device, to create small punctures on the scalp. These microtraumas, which are usually generated within the upper layers of skin specifically, the epidermis and dermis stimulate the body’s natural wound healing process [32,65,66,67,68,69,70,71].

Microneedling triggers increased blood flow to the affected areas, enabling additional nutrients and oxygen to reach hair follicles and promoting healthy hair growth. The increase in blood flow also stimulates dermal papilla and hair follicle stem cells, encouraging them to produce new hair cells and activating the release of various growth factors involved in the healing process, including platelet-derived, epidermal, and vascular endothelial growth factors. These growth factors are essential for the regeneration of new skin cells, the formation of collagen and other connective tissue components, the formation of blood vessels, the development of new hair follicles, and the production of hair from existing follicles. This, in turn, yields visible improvements in hair quality, density, and thickness [65,66,67,68,69,70,71].

Microneedling is also believed to work by activating cellular regeneration via genetic signaling, including the VEGF and Wnt/β-catenin signaling pathways, leading to angiogenesis, collagen induction, tissue rejuvenation, and follicular vascularization. Hair follicle neogenesis has also been shown to occur via the Wnt signaling pathway following wounding and wound healing. Wnt/β-Catenin signaling is important for hair follicle regeneration, hair shaft growth, and the initiation and maintenance of hair morphogenesis. Microneedling can produce modest improvements in hair growth when used as a monotherapy. However, when used as an adjunct hair loss treatment, it can produce much greater improvements in hair regrowth [32,65,66,67,68,69,70,71].

#### 3.4.1. Microneedling as an Adjunct Treatment

In clinics, microneedling is rarely used as a monotherapy to treat androgenetic alopecia. Instead, it is more often used as an adjuvant therapy and combined with topical medications, like minoxidil or other topical hair loss products. The microchannels created through microneedling are believed to improve the transdermal penetration of these topicals, allowing for higher concentrations to reach the vascularized dermis directly, and therefore increasing their effectiveness, for example, in the case of non-responders to FDA-approved treatments such as minoxidil and finasteride. Between 50 and 75% of non-responders to 5% minoxidil and oral finasteride have achieved improvements in hair regrowth after adding microneedling to their hair loss treatment routines [72].

Most notable example of improved effectiveness has been a comparative study of 100 men with androgenetic alopecia that were split into two groups. The first group received the standard FDA-approved minoxidil treatment, while the second received minoxidil along with microneedling. The addition of microneedling resulted in an increased hair count of 91.4 follicles per cm^2^ compared to that in the minoxidil monotherapy group, whose hair count increased by just 22.2 follicles per cm^2^ [32]. While the results involving the addition of microneedling always show an improvement, they are not always as dramatic. A similar study assessing minoxidil and the combination of minoxidil and microneedling showed an increased hair count of 12.82 hairs per cm^2^ in the minoxidil and microneedling group, compared to an increase of 1.89 ± 8.94 in the group using minoxidil on its own [73].

Microneedling has also been shown to help improve healthy hair growth in other studies. A study involving three treatment groups—minoxidil, minoxidil plus platelet-rich plasma treatments, and minoxidil, platelet-rich plasma treatments and microneedling—found that hair strength was best in the tri-therapy-treated men, followed by those who had received the di-therapy. Nearly twice as many men (87.1%) who received the tri-therapy treatment retained hair following a hair pull test compared to those who received minoxidil monotherapy (48.4%) [67]. To date, no microneedling device has obtained clearance for the treatment of hair loss. While over a dozen microneedling devices have been cleared for use by the FDA, all of them have been cleared for cosmetic dermatological uses, such as wrinkle and scar reduction.

#### 3.4.2. Delivering LLLT via Microneedling

Maintaining the safety profile of an LLLT-based hair loss treatment, while improving effectiveness, would substantially impact the hair loss treatment industry, particularly given the large number of non-responders to current FDA-approved solutions. The primary challenge of this technique is the need to deliver enough energy to the dermis to achieve an effect, often thermal, without causing severe disruption that could lead to burns. Several researchers have demonstrated how the blood vessels taken from animal models could be dilated using delivery of light in the red spectrum (from 630 to 700 nm wavelength). The light fluences shown to be most effective for dilation ranged from 5 to 10 J/cm^2^ when irradiated directly on the vessel surface [74,75,76].

It is possible to bypass the skin’s barrier in a controlled fashion. One can create a hole or another type of opening through surgery, or create many holes simultaneously in a minimally invasive manner via microneedling. Combining lasers with a technique such as microneedling would allow light to bypass the skin’s barrier in a controlled manner that can be used to deliver stimulation therapies.

Based on prior optical simulations, only 5% of the light applied to the skin’s surface reached a depth of 2 mm, where the majority of blood vessels and capillaries of the hair follicles reside. Approximately 90% of light loss typically occurs in the epidermis due to the skin’s melanin layer, found less than 500 μm deep [40]. However, inserting a light source into the skin by as little as 500 microns can increase light delivery by an order of magnitude or more, specifically, the desired 2 mm depth. This is the premise of a novel device for AGA developed using the concept of precision LLLT, which is currently being tested in clinical trials [77].

**Figure 3:**
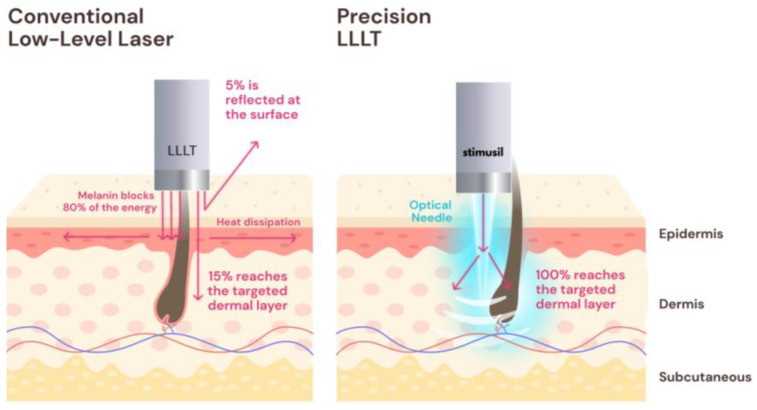
Skin penetrance using a conventional LLLT device versus precision LLLT.

Combining microneedling devices and topical minoxidil has shown success [72]. Precision LLLT utilizes a novel approach to photobiomodulation delivery, which is intended to reproduce the success of combining minoxidil with microneedling without the use of pharmaceuticals. This concept applied using the SAGA-001 device combines microneedling and laser light delivery for hair growth. This device uses lasers injected into the skin’s dermis to increase light penetration and the effectiveness of LLLT treatments. Microneedling utilizes mechanical damage to induce a wound healing effect, while LLLT utilizes red to near-infared light to stimulate vascular dilation, cell proliferation, and regeneration [78,79]. The SAGA device pierces through the epidermis using novel optical microneedles that bypass the melanin layer in order to safely and efficiently deliver more energy to the hair follicle, as shown in Figure 3. Red, pulsed lasers are used to deliver the maximum amount of stimulatory energy without causing photothermal damage, remaining a safer, purely photostimulatory regime [77].

In mid-2023, StimuSIL initiated a pivotal human study testing two versions of the SAGA-001 device on men with androgenetic alopecia. The participants are males aged from 22 to 55 years old with Hamilton–Norwood stage IIa-V hair loss and Fitzpatrick Skin Types I-IV. The study aims to confirm the safety and effectiveness of two versions of the SAGA-001 device in treating male pattern hair loss. Unlike most hair loss treatments that require the users to apply or take medications daily, or use devices multiple times a week, SAGA-001 treatments are only required every two weeks. This means that participants in this 24-week clinical trial receive a total of 12 treatments. The study will quantify results by assessing changes in terminal hair density and hair thickness at 16 and 24 weeks through use of global and macro photography [77].

## 4. Discussion

**Figure 4:**
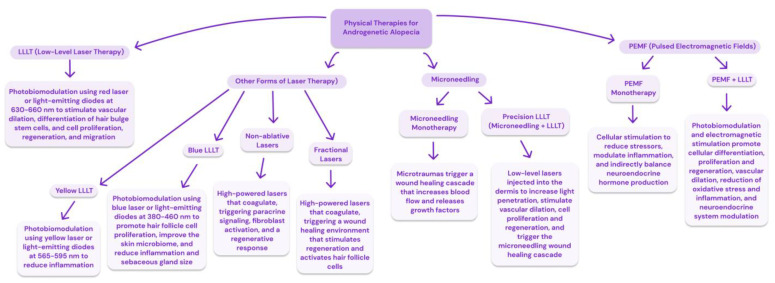
A summary of physical therapies available for the treatment of androgenetic alopecia.

**Figure 5:**
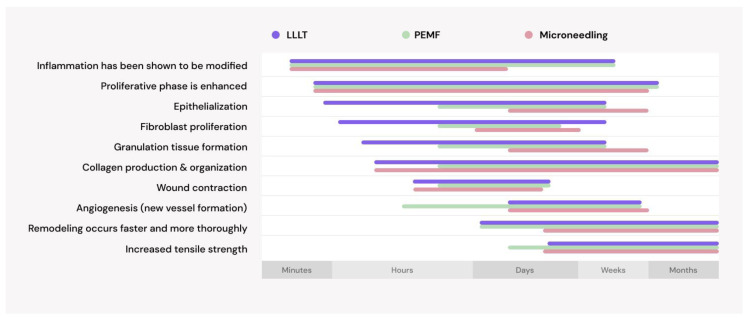
Similar pathways are activated after administering LLLT [80,81,82,83,84,85,86,87,88,89,90,91], PEMF [92,93,94,95,96,97,98,99,100,101,102,103,104,105,106,107,108,109,110,111,112,113,114], and microneedling [31,32,48,49,115,116,117].

Physical therapies seem to be promising treatments for AGA, with various options available for individuals to use at home as well as in clinical settings, as summarized in Figure 4. These treatments all work directly on the hair follicle, producing improvements with a low risk of side effects. In fact, these physical therapies act across the same pathways, simply taking effect at different stages, as shown in Figure 5.

Photobiomodulation, especially LLLT, has been widely studied for its beneficial effects on wound healing and its ability to increase the production of ATP, ROS, and NO, which are essential for cellular energy, signaling, and vascular functions, respectively. LLLT helps modulate the inflammatory response, reducing the duration and intensity of inflammation, thus creating a more conducive environment for healing. LLLT also supports a number of proliferative activities, stimulating fibroblast proliferation, granulation tissue formation, collagen production, epithelialization, and remodeling, which significantly accelerate the healing process. These beneficial effects are not necessarily limited to the wound itself, as LLLT may produce effects distant from the immediately treated area [80,81,82,83,84,85,86,87,88,89,90,91].

While it is less well studied, Figure 5 shows that PEMF has very similar mechanisms of action and works along the same pathways as LLLT. Like LLLT, PEMF has been shown to modulate inflammatory responses, promote proliferative activities, enhance cellular metabolism, improve blood flow, and encourage the release of growth factors and cytokines necessary for wound healing [92,93,94,95,96,97,98,99,100,101,102,103,104,105,106,107,108,109,110,111,112,113,114]. Microneedling induces controlled microtrauma, directly implicating it in the wound itself. However, by initiating an intentional inflammatory response, it sets the stage for subsequent healing that ultimately use the same mechanisms of action as LLLT and PEMF [31,32,48,49,115,116,117].

As the only physical therapy cleared for the treatment of AGA by the US FDA, the vast majority of the literature on physical treatments for this type of hair loss focuses on red low-level light therapy. However, there is a notable and substantial amount of variability reported in LLLT study parameters, which range from 8-min treatments three times per week to 18-min treatments once a day. Notably, the 8-min treatments using the HairMax Laser Comb showed an increase of 26 terminal hairs/cm^2^ after 26 weeks compared to an increase of 6 total hairs/cm^2^ after 24 weeks using the Restore ID-520 for 30 min, three times per week [24,25]. This dose-dependent response is indicative of hormesis and may be relevant to consider for all the physical therapies, given that they involve similar regenerative pathways, as shown in Figure 5.

To date, the only available options for home-use physical treatments are LLLT devices, which are available as comb/brush or cap/helmet type devices [21]. These products have been proven as safe and effective tools for the treatment of hair loss. There are currently no FDA-approved blue or yellow LLLT devices, and all the other forms of laser hair therapy must be administered in a clinical setting. The studies on these alternative forms of laser hair therapy remain very limited. However, of note is the Er/G laser, which reported an increase of 57 total hairs/cm^2^ after 20 weeks of treatment (10 treatment sessions) [38]. This study applied significantly fewer treatments and attained approximately 3–4 times as much hair growth compared to what has been reported by some LLLT studies. It will be of great interest to see if further studies exploring the use of nonablative lasers for hair loss will be able to produce comparable results.

A wide variety of microneedling devices are sold over the counter and can be used by individuals at home. However, microneedling treatments for hair loss are generally the most successful and have a much lower risk when performed in a sterile clinical setting. Furthermore, this physical therapy is best used as an adjunct treatment. It is still unknown how effective combining microneedling with most other physical therapies would be. The first study of this kind, combining microneedling with LLLT, is still in clinical trials [77].

Thus far, the studies exploring PEMF as a treatment for AGA have been minimal. However, the limited research on this subject shown in Table 1 indicates that PEMF may very well produce results that are comparable with LLLT. In fact, PEMF may very well have the potential to be a superior hair loss treatment as, when used as a monotherapy, the reported increases in terminal hair growth are comparable to the improvements in total hair growth reported in some LLLT studies [35,36]. Multimodal approaches reporting improvements in total hair growth indicate that the addition of PEMF produces enhanced outcomes [33,34], implying that there may be a synergistic effect or a novel mechanism of action on the hair follicle. Notably, the administered treatments listed in Table 1 utilize devices that are only appropriate for use in clinical settings. However, given the array of other indications safely utilizing PEMF, it will be interesting to see if future research utilizes devices for AGA that can be implemented in home-use settings.

## 5. Conclusions

Several physical treatments show promise for the management of AGA. In particular, low-level laser therapy demonstrates significant potential due to the photobiomodulation effects on mitochondria, which can enhance cellular energy production and potentially stimulate hair growth. The other light sources, including fractional and nonablative lasers, as well as blue and yellow light therapies, offer a range of options that are becoming increasingly popular treatment options for AGA, particularly as adjunct treatments for individuals who are non-responsive to FDA-approved solutions.

Pulsed electromagnetic field therapy is another modality that has been explored for AGA, with the studies indicating its efficacy not only as a standalone treatment, but also in combination with other therapies. Multimodal approaches that include PEMF have shown enhanced outcomes, implying a synergistic effect or a novel mechanism of action affecting the hair follicle, which should be explored further in future research.

Microneedling is primarily recognized for its adjunctive role in the treatment of AGA. By creating micro-injuries to the skin, microneedling facilitates the deeper penetration of other hair loss therapies, potentially increasing their effectiveness. The integration of LLLT delivery via microneedling devices is a novel approach that combines the mechanical and photonic stimulation of hair follicle stem cells, which could revolutionize the treatment protocols.

The benefits and efficacy of multimodal physical treatments for AGA is of great interest as the side effects of these devices are minimal, making their combined potential both highly effective and low-risk. Future research should focus on optimizing treatment parameters, exploring the synergistic effects of multimodal approaches, and establishing long-term efficacy and safety profiles for these physical therapies. It is crucial for the upcoming studies to continue to explore these combinations in clinical settings, with a focus on identifying treatment parameters that maximize patient outcomes. Once the protocols using these minimally invasive, non-pharmacological treatments are optimized, they will provide substantial relief to those suffering from AGA, enhancing both hair growth and quality of life for affected individuals.

## Data Availability

The data generated and analyzed during this study are included in this published article.
